# Usability Study of Augmented Reality Visualization Modalities on Localization Accuracy in the Head and Neck: Randomized Crossover Trial

**DOI:** 10.2196/75962

**Published:** 2026-01-13

**Authors:** Yao Li, Gijs Luijten, Christina Gsaxner, Kim Grunert, Alexis Bader, Frank Hölzle, Rainer Röhrig, Matías de la Fuente, Jan Egger, Kunpeng Xie, Behrus Hinrichs-Puladi

**Affiliations:** 1 Department of Oral and Maxillofacial Surgery, University Hospital RWTH Aachen, Pauwelsstrasse 30, Aachen, 52074, Germany, 49 2418088231; 2 Institute of Medical Informatics, University Hospital RWTH Aachen, Aachen, Germany; 3 Institute for Artificial Intelligence in Medicine (IKIM), Essen University Hospital, Essen, Germany; 4 Center for Virtual and Extended Reality in Medicine (ZvRM), Essen University Hospital, Essen, Germany; 5 Institute of Computer Graphics and Vision (ICG), Graz University of Technology, Graz, Austria; 6 Department of Systems Design Engineering, University of Waterloo, Waterloo, ON, Canada; 7 Chair of Medical Engineering, RWTH Aachen University, Aachen, Germany

**Keywords:** mixed reality, computer-assisted surgery, visualization techniques, human-machine interface, preoperative planning

## Abstract

**Background:**

Augmented reality head-mounted displays could overcome the spatial dissociation between medical imaging and the surgical field, which may be particularly important in anatomically dense regions, such as the head and neck. Although many head-mounted displays offer markerless inside-out tracking at a fraction of the cost of navigation systems, their overlay accuracy with superimposition (SI) modality onto the surgical field remains limited. The virtual twin (VT), displaying holography adjacent to the surgical field, may offer a viable alternative. However, its performance is still unclear.

**Objective:**

This study aimed to compare the accuracy and efficiency of the two visualization modalities, SI and VT, for anatomical localization in the head and neck region.

**Methods:**

In a randomized crossover trial to compare two augmented reality visualization modalities (SI and VT), 38 participants used a HoloLens 2 to localize point, line-based, and volume-based anatomical structures on head phantoms. Their performance was evaluated with respect to accuracy, workload, time, and user experience.

**Results:**

SI achieved significantly better point localization accuracy than VT both in absolute (mean 14.4, SD 4.2 mm vs mean 15.8, SD 5.5 mm; *P*=.003) and relative accuracy (mean 3.4, SD 2.2 mm vs mean 6.0, SD 5.0 mm; *P*<.001). In line-based structures, accuracy was comparable between SI (average surface distance [ASD], mean 23.4, SD 4.1 mm; Hausdorff distance [HD], mean 31.5, SD 7.8 mm) and VT (ASD=mean 23.0, SD 4.5 mm; *P*=.51; HD=mean 31.0, SD 7.5 mm; *P*=.57). However, SI showed significantly higher deviation than VT in volume-based structure (ASD=mean 37.1, SD 13.8 mm vs mean 34.1, SD 14.2 mm; *P*=.01; HD=mean 52.0, SD 16.8 mm vs mean 49.1, SD 15.8 mm; *P*=.03). Participants were faster with SI (*P*=.02), while workload NASA-TLX (National Aeronautics and Space Administration Task Load Index) scores did not demonstrate a significant difference (*P=*.79).

**Conclusions:**

Given that SI did not clearly outperform VT under overlaid soft tissue and viewing challenges, VT remains a viable alternative in certain surgical scenarios where high accuracy is not required. Future research should focus on optimizing viewing angle guidance and the linkage between the anatomical target and the skin surface.

## Introduction

The head and neck region contains a variety of complex anatomical structures, including numerous vital nerves, blood vessels, and organs [1]. Accurate localization of these anatomical structures is crucial in surgical practice to minimize deviation and improve outcomes [2]. Conventional medical imaging techniques, such as computed tomography (CT) and cone beam CT, as well as magnetic resonance imaging, are primarily used for diagnosis and preoperative planning [3,4]. Medical images require surgeons to mentally map medical images onto the patient’s anatomy during the operation. This process demands a high level of cognitive effort, especially in the anatomically dense head and neck region, where misinterpretation could compromise the surgical accuracy and outcomes [5,6]. Surgical navigation systems (SNS) offer solutions by integrating image data into the surgical workflow. However, the limitations of the 3D display still leave the operator reliant on spatial imagination to understand complex anatomy. Furthermore, the broader adoption of SNS has been impeded by high expenses, the inherently sophisticated configurations like optical tracking cameras and reflective markers, and the possible additional radiation exposure to patients and staff [7,8]. As a result, there is still a lack of a cost-effective, intuitive, 3D interactive visualization approach that seamlessly displays the patient’s medical images in the field.

Augmented reality (AR) could fill this gap by providing real-time holographic images directly within the surgical field mainly through head-mounted displays (HMDs) [7,9]. Moreover, many current AR HMDs can provide markerless inside-out tracking at a fraction of the cost of SNS and eliminate the need for additional markers [10,11]. Unlike SNS, which typically tracks the patient and instruments, this kind of HMD-based tracking focuses on aligning virtual content with the patient’s anatomy to enable hologram overlay. However, the overlay or registration accuracy of many HMDs is still not as accurate as traditional SNSs with external optical tracking at the millimeter level [10]. This limitation becomes particularly critical for the superimposition (SI) visualization modality, where virtual anatomical structures need to be precisely placed on real anatomy, a process referred to as registration [12-14]. In addition, SI may introduce occlusion, as holograms can obstruct the surgeon’s view of anatomy or instruments. These challenges raise concerns about the feasibility of SI as the optimal visualization modality for AR-assisted surgery, given the setup of currently available HMDs free of external tracking [15].

An alternative visualization modality is the virtual twin (VT), where the holographic representation is displayed adjacent to the physical anatomy instead of directly overlaid on the anatomy [15,16]. By avoiding overlay, VT reduces dependence on registration accuracy and eliminates occlusion.

However, the accuracy between two modalities under markerless HMD-based tracking remains unexplored. Yet, this could be important, since if SI with intrinsic markerless tracking does not show any advantage over VT, then VT would be the favored modality for certain surgical scenarios. Therefore, the aim of this crossover randomized controlled trial (RCT) was to compare the accuracy and efficiency of the two visualization modalities, SI and VT, for anatomical localization in the head and neck region. Localization accuracy was assessed on phantom heads for clinically relevant targets, including nerve exit points, the inferior alveolar nerve, and the salivary glands. Task duration and subjective workload were evaluated as secondary endpoints.

## Methods

### Overview

In total, 38 participants with different professional backgrounds (dental and medical students, resident and specialist surgeons in oral and maxillofacial, oral, and plastic surgery) were recruited and performed drawings on polystyrene foam head phantoms (Model SAM, Friseurbedarf D. M. Rudolph) in a crossover RCT with SI and VT visualization modalities. The participants were asked to draw the structures on the head phantoms, wearing HoloLens 2 (HL2; Microsoft Corp). The primary endpoint was the localization accuracy of the anatomical points (0D), which encompass nerve exit points at the supraorbital, infraorbital, and mental foramina. Secondary endpoints included the delineation accuracy of the inferior alveolar nerve pathways (2D) and salivary glands (parotid and submandibular; 3D), cognitive workload, and user experience.

### System Description and Implementation

The AR visualization software for the HL2 was developed in-house to display anatomical 3D models in relation to the physical anatomy of patients or phantoms. Within the application, switching between the two different visualization modalities for the 3D models was possible. In addition to the HL2 software, a pipeline processed the medical image data. This pipeline converted volumetric CT scans into 3D models optimized for interventional planning and efficient rendering on the HL2.

Based on these requirements, the planning pipeline was built to segment the structures into meshes in 3D Slicer (version 5.2.2; The Slicer Community). The structures comprising the skull, salivary glands, and nerve exit points were manually segmented from a publicly available head and neck CT dataset [17], while the inferior alveolar nerves were segmented from a nonpublic dataset from the Medical University of Graz. A head phantom mesh was scanned by the Artec Leo 3D scanner (Artec 3D) as the skin surface. Finally, all segmented anatomical structures were nonlinearly registered to the scanned skin surface.

Our AR application was developed using Unity (version 2022.3.6f1; Unity Technologies). The registration between the head phantoms and the virtual head was implemented using the Vuforia software development kit (version 10.16.5, Parametric Technology Corporation). Vuforia Engine is a cross-platform AR solution that offers a variety of tracking features, which was frequently used in research for AR registration in surgical scenarios [18-20]. The model targets (object tracking) were applied, which possibly used edge-based techniques (not revealed by Vuforia) to recognize and track objects in real-time [21]. First, the scanned head model was uploaded to the model target generator tool and configured into a model target that could be integrated into Unity. After the software was deployed to the HL2, the Vuforia engine initiated tracking for target alignment. Once the participant is satisfied with the alignment, she or he could lock the tracking to anchor the virtual model in the environment. Similarly, in VT, Vuforia would track the phantom, and then the model would appear next to it; locking the tracking again would fix the model in place. The hand menu assisted users in controlling the visibility of various anatomical structures, including the skin and target structures. In addition, sliders were implemented to allow real-time adjustment of the transparency and brightness of these structures.

### Trial

**Figure 1.**
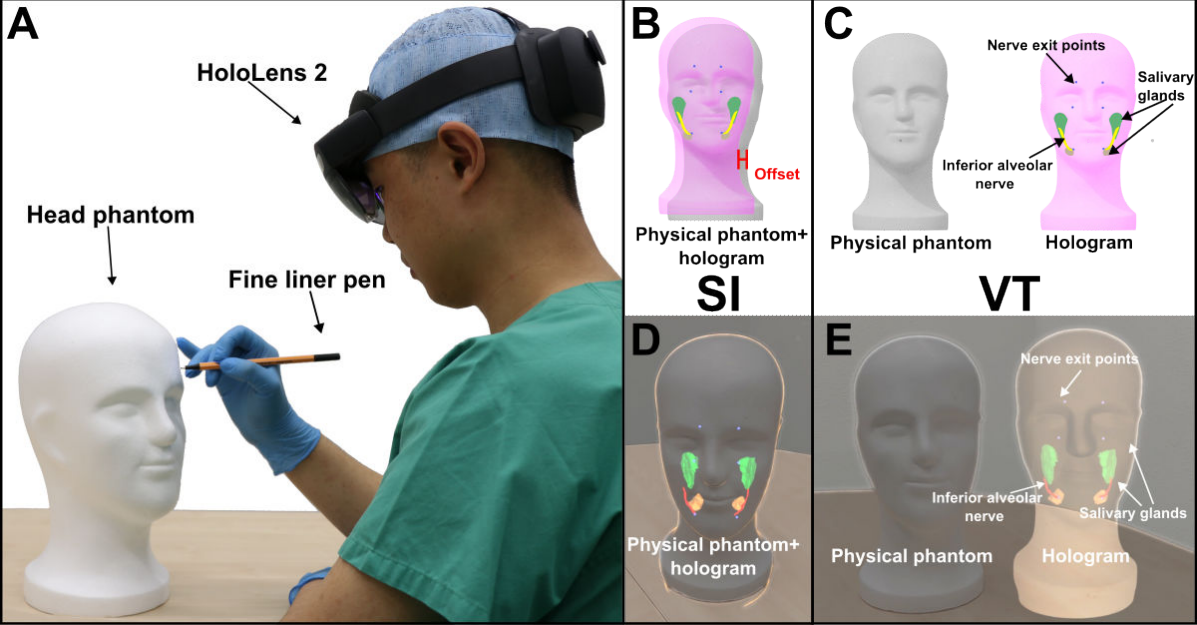
Illustration of two augmented reality visualization modalities using HoloLens 2. (A) Participant drawing anatomical structures (nerve exit points, inferior alveolar nerves, and salivary glands) on the polystyrene head phantom with HoloLens 2. (B) Schematic illustration of SI showing physical and holographic alignment with potential rigid offset and occlusion. (C) Schematic illustration of virtual twin showing how holograms are displayed free of misalignment and occlusion problem. (D) SI modality in HoloLens 2, where holograms were overlaid directly into the physical head phantom. (E) Virtual twin modality in HoloLens 2, where the holograms were displayed spatially adjacent to the physical head phantom. SI: superimposition; VT: virtual twin.

The participants were asked to fill out the initial questionnaire, which included demographic information (age, gender, educational stage or professional experience, professional field, and prior experience with AR and HL2). Randomization was generated by BHP using a randomized allocation rule to determine the starting modality (sequence) and the side of the face (right or left). The experiment assistant (KG) enrolled and assigned participants to the sequence of intervention. Registration was done once at the beginning of each modality by the experiment assistant, who could lock or unlock the tracking for registration as needed. Subsequently, they wore HL2, ran the eye calibration, and received a brief introduction to the device and the user interface with the 2 modalities. During this short session, they familiarized themselves with the device and its functions. The entire familiarization process was completed in less than 3 minutes, although precise timing was not recorded. Participants were then instructed to delineate target anatomical structures on the head phantom surface using Point 88 fine liner pens (Stabilo; Figure 1). This task was performed on the assigned half of the face using the first modality, with time recorded via a stopwatch. Upon completion, participants filled out the Likert questionnaire and NASA-TLX (National Aeronautics and Space Administration Task Load Index) for that method and an open-ended questionnaire. The same procedure was then repeated on the other side of the face using the second modality, followed by the corresponding questionnaires. Finally, an open-ended questionnaire for preference was answered.

### Evaluation

**Figure 2.**
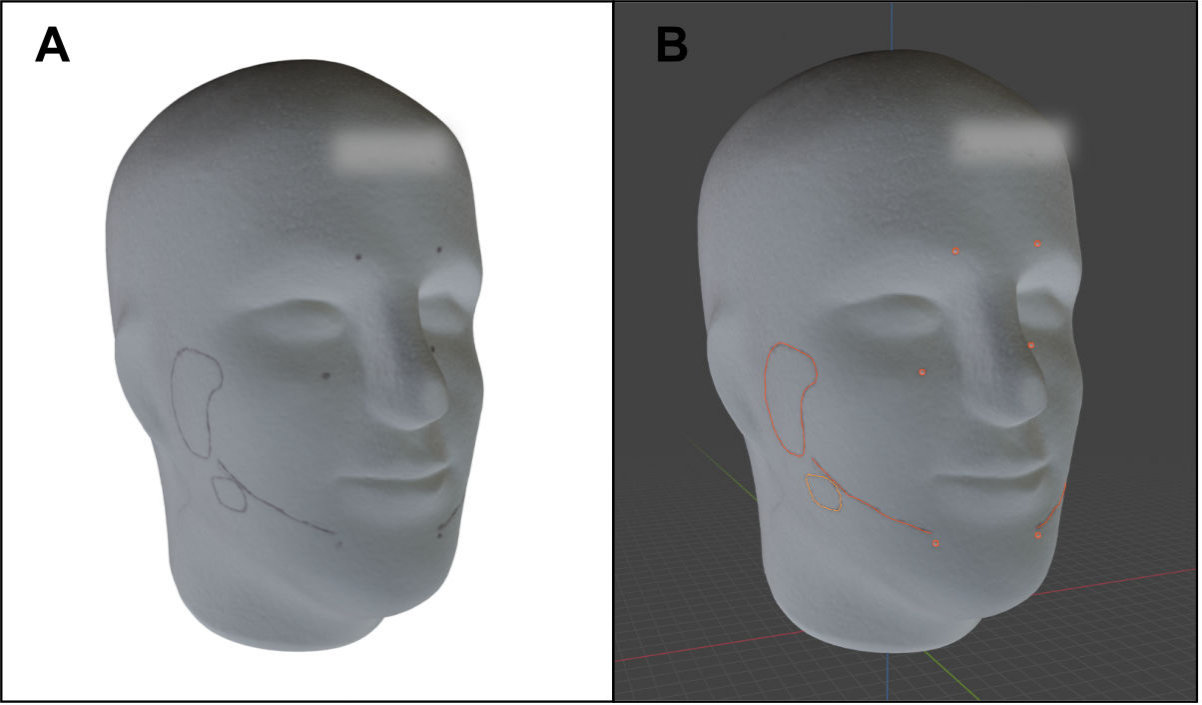
(A) Scanned polystyrene head phantom with delineation. (B) In Blender, the scanned polystyrene head phantom is shown with inferior alveolar nerve and salivary glands annotations, and spheres marking the nerve exit points (orange).

After the trial, all the polystyrene head phantoms were scanned with the Artec Leo 3D scanner (Figure 2). To enable comparison, all head phantoms with the participant’s delineations were registered to the virtual planned head in a pipeline by a Python (version 3.10; Python Software Foundation) script. The two-stage pipeline was initiated with a global random sample consensus alignment, followed by a local refinement with point-to-plane iterative closest point, achieving <0.4 mm root mean square error. Two independent investigators (YL and KG) evaluated the scanned heads using Blender (version 4.2; Blender Foundation). Both investigators were blinded to the applied visualization modality. To minimize a possible recall bias, KG, who served as the experiment assistant during data acquisition, underwent a washout period of 2 months before participating in the blinded evaluation. Nerve exit points were drawn by the stroke points and placed spheres. The nerve paths and salivary glands were drawn by the grease pencil tool along the curves on the head phantom surface, and the strokes were transformed into meshes in Blender (Figure 2).

Afterward, the points and curves were automatically compared using a Python script. The analysis for nerve exit points (0D) involved calculating Euclidean distance, which is the shortest distance in 3D space between the planned and drawn points, and we referred to this as absolute accuracy. Relative accuracy, defined as landmark-to-landmark localization accuracy, was compared by the Euclidean distance between the supraorbital-infraorbital and infraorbital-mental foramina on drawn versus planned landmarks. Since all anatomical targets were located on the underlying bone, yet localization was performed on the phantom’s external surface, the concept of soft-tissue thickness was additionally introduced to capture the distance between the target structures and the skin. It was defined as the shortest distance from each anatomical point (0D) to the surface and as the mean of the vertex-to-surface distances for 2D nerve pathways and 3D salivary glands. Furthermore, the Hausdorff distance (HD) and the average surface distance (ASD) were used in order to assess the alignment and accuracy of the contours of the nerve paths (line, 2D) and salivary glands (volume, 3D). HD captures the maximum of the minimum distances between the two surfaces, providing insight into the worst-case alignment error, while the ASD quantifies the mean discrepancy, reflecting the overall degree of alignment.

The Likert questionnaire and NASA-TLX were quantitatively analyzed to assess usability and perceived workload. In addition, the feedback from open-ended questions was summarized by YL and reviewed by BHP.

### Sample Size Calculation

The sample size calculation was conducted in R software (version 4.3.1; R Foundation for Statistical Computing). A minimum effect size of 5 mm was established as the threshold for an acceptable difference between the two modalities in absolute accuracy. A 5 mm difference in absolute accuracy causes a surface discrepancy exceeding 5 mm due to the geometric relationship, making it clinically relevant and detectable by oral and maxillofacial surgeons, corresponding to the widely accepted minimum margin in head and neck oncologic surgery [22,23]. Based on the results of a pretrial with 4 participants, the mean absolute accuracy was 10.1 (SD 4.8) mm (SI) and 12.1 (SD 5.0) mm (VT) across all nerve exit points. A normal distribution of the pretest values (Shapiro-Wilk test; *P*=.70) resulted in a required number of cases of 34 for the unpaired *t* test. An additional 4 participants were included to compensate for nonevaluable datasets and for dropout or withdrawal of consent.

### Statistical Analysis

Statistical analysis was also performed in R. A linear mixed-effects model (LMM) was applied using the *lmerTest* package [24]. This LMM assessed the absolute accuracy at the point structures, modalities (SI vs VT), the sequence (starting method), the group (dental and medical students, and surgeons), subcutaneous soft tissue thickness, and side (left or right) as fixed effects and the participants as a random effect. When analyzing the ASD and HD for line and volume-based structures, the same LMM framework was applied. Subcutaneous soft tissue thickness was specifically included to account for anatomical variation across different locations. However, it was not considered in the analysis of relative accuracy for point structures, which instead relied more on spatial reference to other anatomical landmarks.

The normality of the data distribution was assessed using the Shapiro-Wilk test. Duration and each Likert question between methods were compared using the Mann-Whitney *U* test. The NASA-TLX scales were compared by unpaired two-tailed *t* test. For all tests mentioned, a *P* value of <.05 was considered significant.

### Ethical Considerations

This study was approved by the local ethics committee of the University Hospital RWTH (Rheinisch-Westfälische Technische Hochschule) Aachen (EK 24‐127; Chairman Prof Ralf Hausmann; April 3, 2024). The study was registered with a study protocol in advance in the German Clinical Trial Register (DRKS00032835) and followed the CONSORT (Consolidated Standards of Reporting Trials) 2010 guidelines (Checklist 1) and its extension designed and modified specifically for crossover studies, as illustrated by the flow diagram [25,26]. Informed consent was obtained from all participants involved in the study. To protect the privacy of the participants, all participants were anonymized, and no personally identifiable information was stored with the research data. It is to be noted that no financial compensation was provided to the participants involved in the present trial. Nevertheless, as a token of appreciation, two vouchers with a total value of €15 (US $17.5) were distributed through a raffle.

## Results

### Cohort

**Figure 3.**
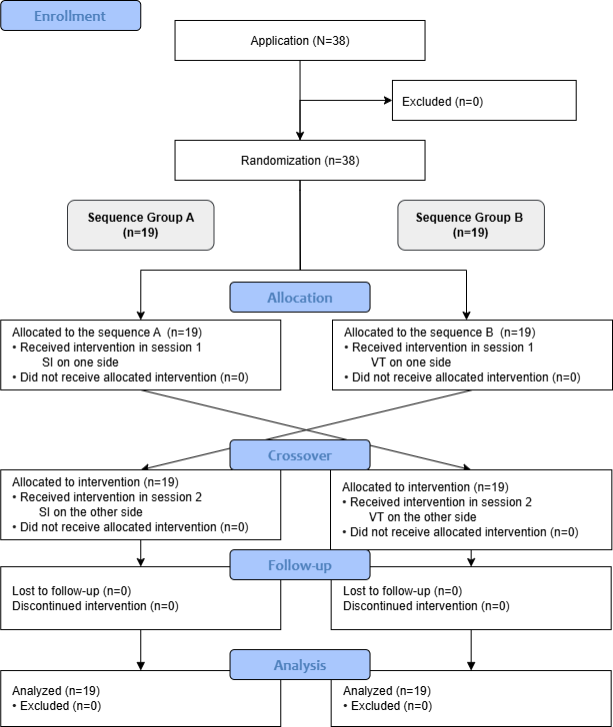
CONSORT (Consolidated Standards of Reporting Trials) flow diagram illustrating the enrollment, allocation, crossover, follow-up, and analysis of participants in the study. SI: superimposition; VT: virtual twin.

A total of 38 participants (16 females and 22 males) were successfully included in the study, comprising two groups, namely surgeons, and medical and dental students following the flow (Figure 3). Among the 18 surgeons, there were 12 residents and 6 specialists. This group included 9 oral and maxillofacial surgeons, 5 oral surgeons, and 4 plastic surgeons. In the student group, which consisted of 20 participants, 17 were dental students and 3 were medical students. The average age of participants was 26.8 (SD 5.1; range 20‐43). The average clinical experience of surgeons was 4.0 (SD 4.3) years, and the average clinical experience of medical and dental students was 4.2 (SD 0.9) years; mean 8.3 (SD 1.6) semesters (Table 1).

**Table 1.** Characteristics of the cohort.

Parameter		Surgeon (n=18)	Student (n=20)	Total (n=38)
Sex, n (%)				
Female		4 (22.2)	12 (60)	16 (42.1)
Male		14 (77.8)	8 (40)	22 (57.9)
Age (years)				
Mean (SD)		30.3 (5.2)	23.8 (2.5)	26.8 (5.1)
Range		23-43	20-30	20-43
Profession, n (%)				
Medical		—^a^	3 (15)	3 (7.9)
Dental student		—	17 (85)	17 (44.7)
Oral surgery		5 (27.8)	—	5 (13.2)
Oral and maxillofacial surgery		9 (50)	—	9 (23.7)
Plastic surgery		4 (22.2)	—	4 (10.5)
Clinical study/work experience (years)				
Mean (SD)		4.0 (4.3)	4.2 (0.9)	4.1 (3.0)
Range		0.0-15.0	3.0-6.0	0.0-15.0
Previous experience with AR (Likert score, 1–5)^b^				
Mean (SD)		2.4 (0.8)	2.1 (1.0)	2.2 (0.9)
Range		1.0-4.0	1.0-5.0	1.0-5.0
Previous experience with HL (Likert score, 1–5)^c^				
Mean (SD)		1.7 (0.8)	1.1 (0.3)	1.4 (0.6)
Range		1.0-3.0	1.0-2.0	1.0-3.0

a Not applicable.

b AR: augmented reality; Likert scores from 1=”never heard of” to 5=”expert.”

c HL: HoloLens; Likert scores from 1=“never used” to 5=“I use it several times a week.”

### Localization Accuracy

**Figure 4.**
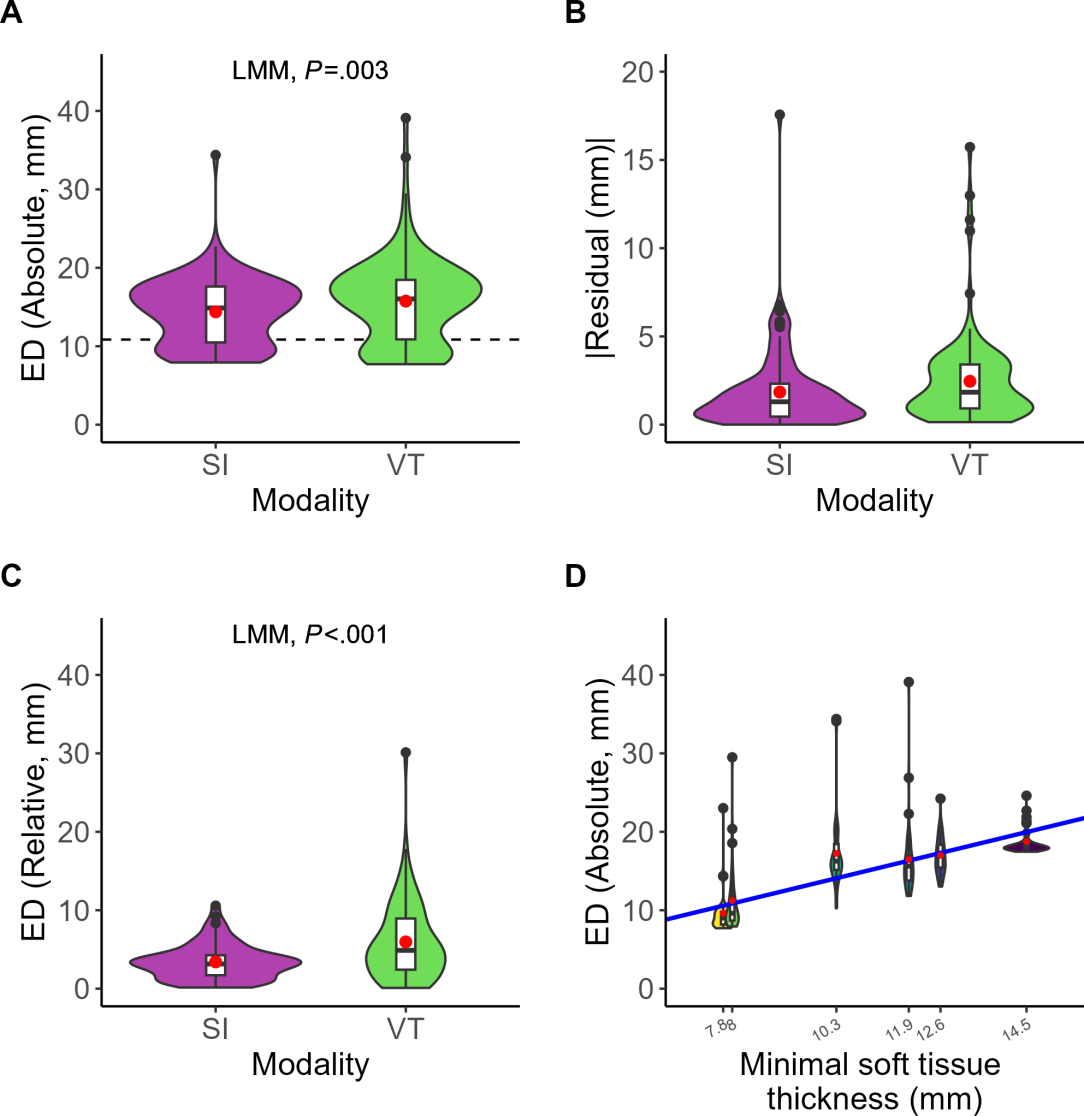
Comparison of localization accuracy (y-axis) at nerve exit points (0D) between superimposition (purple) and virtual twin (green; x-axis). (A) Euclidean distance for absolute accuracy. (B) Absolute residual error from the linear mixed-effects model. (C) Euclidean distance for relative accuracy. (D) Relationship between Euclidean distance for absolute accuracy (y-axis) and subcutaneous soft tissue thickness (x-axis). The solid blue line depicts the fitted linear mixed-effects model regression. ED: Euclidean distance; LMM: linear mixed-effects model; SI: superimposition; VT: virtual twin.

In the 38 scanned head phantoms, all the required structures were successfully delineated, except for 1 pair of nerve exit points at infraorbital foramina and 1 pair at supraorbital foramina, which were missed by a single participant. The absolute accuracy of the nerve exit points (0D) was significantly higher in SI (mean 14.4, SD 4.2 mm) than VT (mean 15.8, SD 5.5 mm), with a mean difference of 1.4 (95% CI 0.5‐2.3; LMM; *P*=.003) mm. The absolute accuracy was correlated with the soft tissue thickness. For each 1 mm soft tissue thickness, the accuracy decreased by 1.4 mm (*P*<.001), while no significant difference was found in sequence (*P=*.84) and group (*P*=.40) as fixed effects in the LMM. The average participant bias was 0.8 (SD 0.8) mm. The mean absolute error of the LMM residuals was 1.8 (SD 2.9) mm for SI and 2.5 (SD 3.5) mm for VT, respectively. The relative accuracy of the points was significantly higher for SI (mean 3.4, SD 2.2 mm) than VT (mean 6.0, SD 5.0 mm) by 2.6 (95% CI 1.3‐3.8 mm; LMM; *P*<.001; Figure 4). In Figure 4, each violin plot (colored) includes a boxplot (white), with a red dot indicating the mean value. The black points represent the outliers. The dashed line marked the average subcutaneous soft tissue thickness over the nerve exit points.

**Figure 5.**
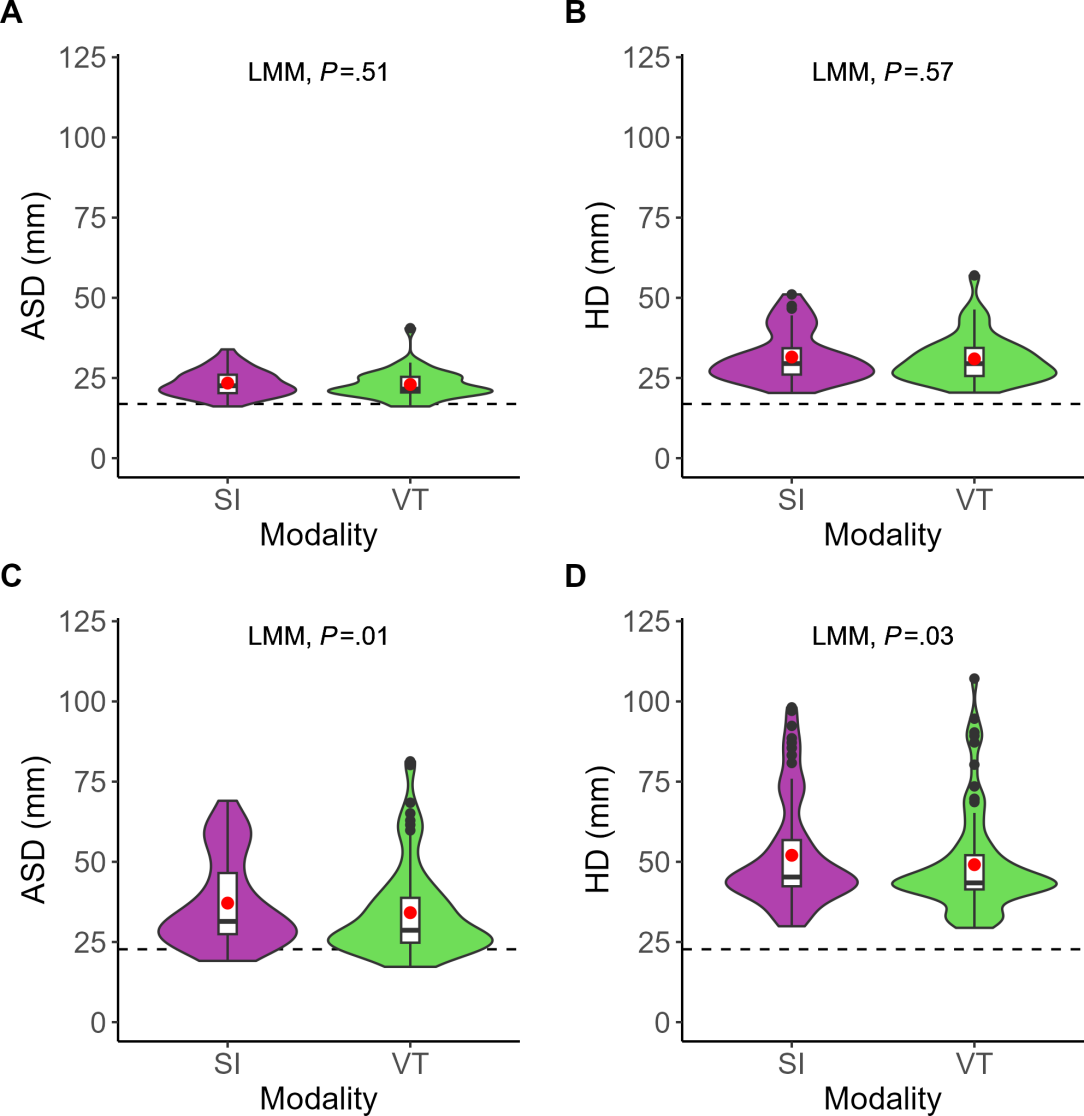
Comparison of localization accuracy (y-axis) for inferior alveolar nerve pathways (2D) and salivary glands (3D) between superimposition (purple) and virtual twin (green; x-axis). (A) Average surface distance for inferior alveolar nerve pathways (2D). (B) Hausdorff distance for inferior alveolar nerve pathways (2D). (C) Average surface distance for salivary glands (3D). (D) Hausdorff distance for salivary glands (3D). ASD: average surface distance; HD: Hausdorff distance; LMM: linear mixed-effects model; SI: superimposition; VT: virtual twin.

The localization accuracy of the inferior alveolar nerve pathways (2D) assessed with ASD and HD was comparable between SI (ASD/HD=mean 23.4, SD 4.1 mm/mean 31.5, SD 7.8 mm) and VT (ASD/HD=mean 23.0, SD 4.5 mm/mean 31.0, SD 7.5 mm), with no significant difference (ASD/HD=mean difference 0.4 mm, 95% CI −1.0 to 2.0 mm; LMM; *P*=.51/mean difference 0.6 mm, 95% CI –1.6 to 2.9 mm; LMM; *P*=.57). Regarding the salivary glands (3D), the localization accuracy measured with ASD/HD (mean 34.1, SD 14.2 mm/mean 49.1, SD 15.8 mm) for VT was significantly more accurate than SI (ASD/HD=mean 37.1, SD 13.8 mm/mean 52.0, SD 16.8 mm) by ASD 3.0 (95% CI 0.7‐5.4 mm; LMM; *P*=.01) mm and HD 2.9 (95% CI 0.2‐5.8 mm; LMM; *P*=.03) mm (Figure 5). In Figure 5, each violin plot (colored) includes a boxplot (white), with a red dot indicating the mean value. The black points represent the outliers. The dashed line marked the average subcutaneous soft tissue thickness over the inferior alveolar nerves and salivary glands.

### Workload and Time

**Figure 6.**
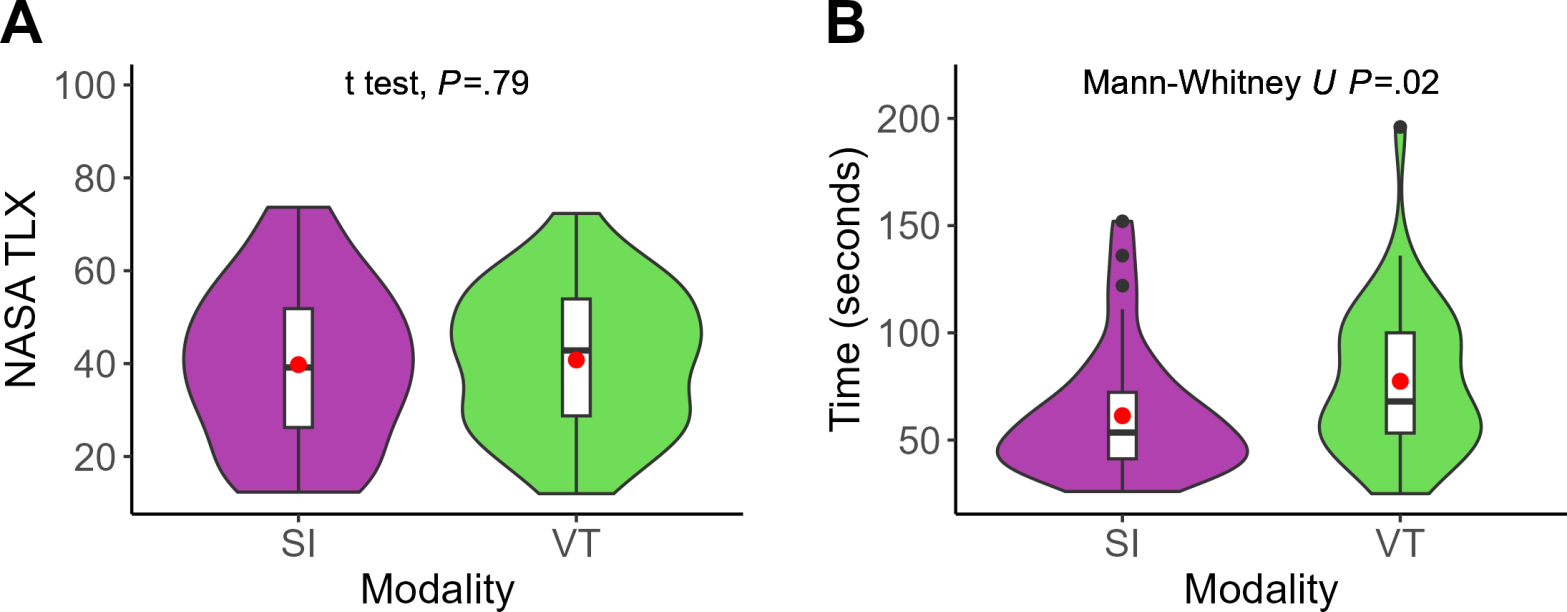
Subjective ratings and task completion time between superimposition (purple) and virtual twin (green) visualizations (x-axis). (A) Subjective workload assessed using NASA-TLX (National Aeronautics and Space Administration Task Load Index) scores (y-axis). (B) Task completion time in seconds (y-axis). NASA TLX: National Aeronautics and Space Administration Task Load Index; SI: superimposition; VT: virtual twin.

The SI method (mean 61.3, SD 29.6 seconds) was significantly faster than the VT method (mean 77.4, SD 34.5 seconds) by 16.1 (95% CI 2.0‐29.0; Mann-Whitney *U* test; *P*=.02) seconds. The NASA-TLX score for the SI method (mean 39.8, SD 17.3) and VT method (mean 40.8, SD 15.2) was comparable, with no significant difference (mean difference 1.0, 95% CI –4.2 to 6.2; *t* test; *P*=.79; Figure 6). In Figure 6, each violin plot (colored) includes a boxplot (white), with a red dot indicating the mean value. The black points represent the outliers.

### Questionnaires

The Likert-type questions (scale 1-4; 1=strong disagreement; 4=strong agreement) showed no significant difference (Mann-Whitney *U* test) between the two modalities (Table 2). The participants perceived no clear advantage in accurate localization of target structures between SI and VT (mean 3.0, SD 0.9 vs mean 3.0, SD 0.6 points; *P*=.61; mean 2.9, SD 0.8 vs mean 2.8, SD 0.7 lines; *P*=.37; mean 2.7, SD 0.8 vs mean 2.7, SD 0.7 volume; *P=*.95). Participants also reported similar levels of confidence (mean 2.7, SD 0.7 vs mean 2.7, SD 0.6; *P=*.84), distraction (mean 2.2, SD 1.0 vs mean 1.8, SD 0.9; *P=*.05), provided assistance (mean 2.9, SD 0.8 vs mean 3.1, SD 0.6; *P=*.46), practicality (mean 2.4, SD 0.9 vs mean 2.8, SD 0.8; *P=*.09), perceived feasibility in interventions (mean 2.8, SD 1.1 vs mean 2.7, SD 0.9; *P=*.57), safety enhancement (mean 2.5, SD 0.9 vs mean 2.7, SD 0.9; *P=*.19), and overall satisfaction (mean 2.8, SD 0.9 vs mean 2.9, SD 0.8; *P=*.66). In addition, positive and negative detailed feedback was provided for both visualization modalities (Table 3). It is noteworthy that 19 participants expressed a preference for VT, 18 participants for SI, and 1 participant expressed equal preference for both.

**Table 2.** Likert questionnaire.

Likert questions	SI^a^, mean (SD)	VT^b^, mean (SD)	Total, mean (SD)	*P* value
I was able to accurately mark the nerve exit points using the (SI or VT) visualization.	3.0 (0.9)	3.0 (0.6)	3.0 (0.8)	.61
I was able to accurately mark the nerve pathways using the (SI or VT) visualization.	2.9 (0.8)	2.8 (0.7)	2.8 (0.7)	.37
I was able to accurately mark the salivary glands using the (SI or VT) visualization.	2.7 (0.8)	2.7 (0.7)	2.7 (0.7)	.95
I was sure where the anatomical structures were located and where to mark them.	2.7 (0.7)	2.7 (0.6)	2.7 (0.7)	.84
I found the using (SI or VT) visualization distracting while marking.	2.2 (1.0)	1.8 (0.9)	2.0 (1.0)	.05
The using (SI or VT) visualization facilitated the localization of anatomical structures in the face.	2.9 (0.8)	3.1 (0.6)	3.0 (0.7)	.46
I found the using (SI or VT) visualization to be practical for use.	2.4 (0.9)	2.8 (0.8)	2.6 (0.8)	.09
I could imagine performing interventions with AR support using (SI or VT) visualization.	2.8 (1.1)	2.7 (0.9)	2.7 (1.0)	.57
I believe that AR^c^ support through (SI or VT) visualization enhances patient safety.	2.5 (0.9)	2.7 (0.9)	2.6 (0.9)	.19
I was generally satisfied with the AR support through the using (SI or VT) visualization.	2.8 (0.9)	2.9 (0.8)	2.8 (0.8)	.66

a SI: superimposition.

b VT: virtual twin.

c AR: augmented reality.

**Table 3.** Summarized open questions.

Visualization modalities	Positive	Negative
Superimposition	3D, intuitive^a^^,^^b^ A novelty experience^a^ Accurate^a^^,^^b^ Beginner-friendly^a^^,^^b^ Clear and detailed^a^ Contrasting colors enhance structural differentiation^b^ Could be observed in all directions^b^ Easy localization of structures^a^^,^^b^ Easy to use^a^^,^^b^ Feeling of safety^b^ Free of time delay^a^ Good guidance and spatial relationship^a^^,^^b^ Inner structures could be easily seen in all directions^a^ Potential to simplify the process^a^ Simple design^a^ Time-saving^a^^,^^b^	Depth is perceived differently in different angles^a^^,^^b^ Difficult to map 3D structures to 3D surface^a^^,^^b^ Hard to identify the position of the structures^a^ Hard to recognize the tip of the pen and place to draw^a^^,^^b^ Have to lock the registration and move the head phantom to fine-tune it to the hologram^a^ Inaccurate overlay, holograms are partially overlaid to the physical head phantom^a^^,^^b^ Need familiarization time^a^ Need to move the head phantom to overlay^b^ Not practical^a^ Relatively lacks sharpness^a^ Restriction of viewpoint, cannot rotate the head phantom to observe after locking the registration^a^ Some structures have merged^a^^,^^b^ The guidance makes the user neglect the critical anatomical landmarks, causing imprecise localization^a^ The hologram is blurred, and the double image is tiring^a^ The position of the head phantom and the participant should be kept constant^a^
Virtual twin	3D visualization, intuitive^a^ Accurate^a^ Assistive setup^a^^,^^b^ Clear visualization of the anatomical structures’ location^a^^,^^b^ Direct views^a^ Easy to use^a^^,^^b^ Good guidance^a^^,^^b^ Guidance to the targets depends on the distance to the landmarks^a^ Head could be moved to draw^a^ Intuitive^a^ Less irritating than SI^b^ Like working with a textbook on the side^a^ No registration problem, 3D model hardly disturbs as an aid^a^ Only exit points are good to paint and recognizable^a^ Opportunity to apply to other structures^b^ With improved guidance, anatomical structures can be localized more effectively, referring to reference only when necessary^a^^,^^b^ Without overlapping, both the pen and the drawing position are clearly visible^a^^,^^b^	Better able to rotate or zoom in^a^ Better to reposition or move the model without moving yourself^a^ Confusing and inaccurate^a^ Deficiency of necessary landmarks^b^ Hard to estimate where to draw^a^ Image lacking sharpness^a^ Lack of 3D guidance^a^ Limited transferability to the real head^b^ Little added value compared to drawing according to anatomical landmarks^a^ Localization cannot be tracked as precisely as with SI^b^ Longer time for eyes to adapt to^a^ Need to turn around the head phantom and hard to find the correct position^a^ Not practical for clinical use^a^ Possible spatial discrepancy, inaccurate drawing, impractical^a^^,^^b^ Required more cognitive effort compared to direct projection^b^ Rotation of the virtual head is restricted^a^ Slower in time^a^^,^^b^ Spatial depth is hard to estimate^b^ Switching attention back and forth between the head and hologram is confusing^a^ The smooth white head phantom offers few points of reference, hard to transfer the anatomical structures^b^

a Dental and medical student.

b Oral and maxillofacial, plastic, and oral surgeons.

## Discussion

### Principal Findings

We systematically evaluated the localization accuracy between two visualization modalities: SI with markerless inside-out tracking and VT for different types of anatomical structures in the head and neck region. The primary endpoint (absolute accuracy of 0D structure) revealed that SI was significantly more accurate than VT by 1.4 mm (*P=*.003). In terms of relative accuracy of 0D point structures, SI also outperformed VT by a margin of 2.6 mm (*P*<.001). VT showed comparable accuracy for 2D structures and notably superior accuracy (ASD, *P=*.01; HD, *P*=.03) for 3D structures, although it required an additional 16 seconds on average (*P=*.02). Likert questions revealed comparable results between two modalities. Feedback from open-ended questions (Table 3) highlighted SI for ease of understanding, intuitiveness, and time efficiency, yet noted persistent challenges with depth perception, visual occlusion, and virtual-real misalignment. Conversely, VT was perceived as simpler, clearer, and free of occlusion and misalignment issues, despite lacking direct positional cues on real head phantoms and requiring frequent attention shifts between physical and virtual models. Overall, user preferences were evenly split, reflecting comparable experiences despite each modality’s distinct strengths and limitations.

### Respective Strengths and Weaknesses

In contrast to VT without tracking, the accuracy of SI depends on the inside-out tracking of the HMD used and can be attributed to 3 main factors, namely the registration accuracy of the tracking (Vuforia), the spatial mapping performance (HL2), and the visual occlusion [27]. Previous studies illustrated Vuforia software development kit’s registration in the HL2 highly depended on the richness of the shape and texture of the tracked target and ranged from less than 2 mm to more than 10 mm for translational error [19,28], which can propagate into an angular deviation of the task-specific cutting plane up to 14.7° [20]. Furthermore, Vuforia tracking is sensitive to environmental light intensity, distance to the target object, and the extent of the surface covered [29]. In addition, HL2 used visual inertial-simultaneous localization and mapping (VI-SLAM) to continuously map the environment and update its position and orientation within a global coordinate system, anchoring virtual content to real-world features [30,31]. However, VI-SLAM’s accuracy can be affected by factors, such as pose prediction latency, user motion, environment, and sensor fusion, such as poor integration between the red, green, blue camera and inertial measurement unit [30,31]. This VI-SLAM error accumulated along the way, reaching 5 mm per 628 mm traveled in the clinical environment [31,32]. Moreover, the jitter latency caused by such sensor fusion could further compromise user experience, increase cognitive load, and induce fatigue [30,33]. Last but not least, visual occlusion, where virtual objects can obstruct or distort the view of the physical counterpart, further compromises the accuracy of SI. Many participants reported difficulty in identifying the position of the pen, drawn line, and occluded virtual content, which was also observed in another study [34]. This occlusion problem could lead to severe damage during surgery by overlooking anatomical structures and events [35,36]. All these factors together may contribute to the overall accuracy achieved by SI.

On the other hand, VT showed comparable accuracy (inferior in 0D, comparable in 2D, and superior in 3D structures) to SI with markerless inside-out tracking of the HMD, but without the aforementioned problems of SI. This was largely due to VT’s design, which bypassed the need for precise virtual-real overlay or accurate anchoring by displaying the virtual model next to the real head phantom. Nevertheless, VT as a visualization modality free of misalignment, unaffected by occlusion, and less sensitive to spatial mapping instability could substitute SI in macro localization tasks. Since VT lacked direct positional cues to guide localization, it likely depended on the surgeon’s ability to estimate distance, where surgeons performed an average error of 1.4 (SD 1.2) mm in 5 mm and 2.0 (SD 1.9) mm in 1 cm estimation in a research [37]. The distances between the nerve exit points in our study were approximately 4 cm between supraorbital and infraorbital foramina and 7 cm between infraorbital and mental foramina. If we assume the estimation error was in a linear model, this corresponded to a mean error of 5.6-9.2 mm, which aligned with VT’s average relative accuracy (mean 6.0, SD 5.0 mm), inferior to SI (mean 3.4, SD 2.2) mm). Therefore, one could argue that SI is only meaningful if its accuracy exceeds the limits of human distance estimation.

### Comparison to Prior Work

In scenarios where precise localization is required, such as orbital fracture reconstruction or trajectory drilling, optical tracking remains the most accurate method to date [15]. Consequently, numerous studies have adopted optical tracking to optimize registration of SI. For instance, Tu et al [38] achieved entry point accuracy of mean 2.8 (SD 1.3) mm and angular accuracy of mean 3.0° (SD 1.2°), optimizing registration accuracy to mean 2.0 (SD 0.7) mm through optical tracking. Similarly, Iqbal et al [39] combined the HL2 built-in camera with an external optical tracking camera, further reducing translation and rotation errors to 2.1 mm and 1.5°, respectively. In contrast, VT with external optical tracking could also visualize both the virtual instrument and the target anatomy but adjacent to the patient in real-time. This framework achieved higher accuracy than the aforementioned SI systems and comparable accuracy to SNS, with translational deviations of mean 0.9 (SD 0.4) mm and mean 1.0 (SD 0.5) mm at entry and end points, respectively, and a rotational deviation of mean 1.1° (SD 0.6°) [15], within the clinically feasible range (~2 mm) [12]. The noticeable difference between VT by 0.9 (SD 0.4) mm and SI by 2.1 mm with a similar optical tracking framework likely resulted from the aforementioned factors, such as registration errors, VI-SLAM instability, jitter, and visual occlusion. This raises the question of whether SI with optical tracking should be considered the optimal AR visualization modality for surgical scenarios, particularly given that VT achieved similar accuracy under similar tracking conditions without encountering these limitations.

However, all these values assume that localization accuracy is measured in anatomically exposed structures, where perfect localization could theoretically reach 0 mm. However, this ignores a crucial aspect of real-world scenarios: anatomical structures are typically covered by tissue, which prevents direct access and inherently limits localization accuracy. In our study, this was particularly relevant due to the soft and bone tissue overlying the anatomical target structures (ie, nerve exit points, inferior alveolar nerves, and salivary glands). According to the literature, the average soft tissue thickness of the head, face, and neck is 9.4 (SD 6.2; range 2.4-28.1 mm) mm in women and 10.5 (SD 7.2; range 2.7-32.4) mm in men [40]. Thus, the measured localization accuracies observed with SI and VT for nerve exit points cannot be directly compared to classical navigation scenarios with fully exposed anatomical targets, as they are composed of 4 main influencing factors


$$Localization\;Accuracy_{ij}=\underset{\text{Anatomical Constraint}}{\underbrace{\beta_{1}\cdot {\text{Overlaying Tissue Thickness}}_{i}}}+$$

$$\underset{\text{Visualization Modality (SI vs. VT)}}{\underbrace{\beta_{2}\cdot {\text{Modality}}_{ij}}}+\underset{\text{Subject Bias}}{\underbrace{u_{j}}}+\underset{\text{Residual Error (AR Noise)}}{\underbrace{\varepsilon_{ij}}}$$

First, the tissue thickness overlaid the target structure. This resulted in a decrease in localization accuracy of 1.4 mm per 1 mm of overlying tissue thickness (LMM, unstandardized coefficient β_1_=1.4; *P*<.001). Then there is the influence of the VT modality, which added an additional error of 1.4 mm compared to SI (LMM, β_2_=1.4; *P=*.003), and the average participant-specific bias, which was 0.8 mm (average magnitude of random intercept for individuals in the LMM). Finally, the residuals described the general pattern of localization error, with a mean absolute error of 1.8 (SD 2.9) mm for SI and 2.5 (SD 3.5) mm for VT.

For line- or volume-based structures, this correlation could not be reliably captured by the model. This was probably because localization accuracy depended not only on the viewing direction but also on dynamic changes in the perceived target margin along that direction. This contrasts with the single-point structure, which is invariably depicted as a point in all directions. As a result, the localization accuracy for line- and volume-based structures is biased by viewing direction and margin variability, in addition to the tissue thickness. As in Van Gestel et al [41], where a brain tumor was dynamically projected onto the skin along a vector from its center to the instrument tip, the participant’s line of sight in our study played a comparable role to the instrument tip. As the viewing angle shifted, the visible margin of the gland changed in real time, introducing variability in the drawn curves and affecting both ASD and HD. Even for targets on the skin, like in wound area estimation using photography, variation in camera angle could introduce 10% error [42]. Although we could not directly quantify viewing angles and changing margins, aligning the participant’s gaze with the vector from the structure’s centroid to its nearest skin projection may help minimize delineation errors related to such bias.

### Clinical Implications

First, VT appears particularly advantageous for tasks requiring coarse localization and stable spatial orientation. VT provides a reliable anatomical context and could help mitigate cognitive errors, such as confusion of lateral sides or anatomical levels. These errors often arise in apparent symmetrical regions, especially in the absence of clear preoperative marking or adequate visual guidance. For example, in thoracolumbar spine surgery, reliance solely on intraoperative fluoroscopy may be insufficient to reliably distinguish vertebral levels, especially in the presence of anatomic variants, inadequate intraoperative imaging fields, and unreliable surface landmarks, with 50%‐67% of surgeons reporting such errors [43,44]. VT could orient the surgeon by allowing the user to align CT-based virtual models with the patient’s posture, enabling clear visualization of the spine and reducing wrong-level or wrong-side misorientation. Second, in maxillofacial reconstruction, the VT technique offers significant value by displaying planned bone segments and prebent fixation plates alongside the operative field. This side-by-side visualization enables real-time comparison and intraoperative adjustment of plate bending, reducing the need for repeated fitting at the surgical site as standard techniques do [45], and thereby lowering the risk of infection. Compared with preoperative 3D printing, such a technique could also minimize fabrication time and offer greater flexibility for intraoperative adjustments. In the following free flap reconstruction procedures, VT offers robustness in environments prone to bleeding, swelling, or tissue deformation, where SI overlays can drift or become unreliable. By anchoring the virtual model generated from virtual surgical planning adjacent to the surgical site, VT provides a stable frame of reference with consistent skeletal landmarks, even when soft tissues shift [46]. Third, VT is well-suited to fractures and postoncologic defects of the orbit and midface requiring symmetry (eg, zygomatic arch, orbital floor, and medial wall) [47]. By rendering the contralateral mirrored anatomy, target orbital volume, planned implant contour, and craniofacial buttresses adjacent to the field, the surgeon could continuously compare the intraoperative reduction with the surgical plan.

While VT may help reduce orientation errors, SI demonstrates its strength in scenarios that demand high-precision localization. For example, in mandibular reconstruction surgery using the anterolateral femoral flap, accurate localization of the perforator vessels is crucial to flap viability and surgical success. One study found the SI with remote-controlled overlay (mean 3.5, SD 2.8 mm) achieved significant superior localization accuracy in anterolateral femoral perforator vessels than ultrasonic color Doppler (mean 9.6, SD 5.8 mm; *P*<.001) [48]. Our findings showed that SI had clear advantages in point-based localization tasks. This feature is particularly important in procedures such as sentinel lymph node biopsy, where accurately identifying nodes just a few millimeters beneath the surface is crucial for surgical success. Duan et al [49] reported that AR SI with motion compensation achieved sub-3 mm localization error in melanoma sentinel lymph node biopsy. Moreover, SI demonstrated significantly superior relative accuracy (*P*<.001). This is because, despite the offset, SI preserved the spatial relationships between landmarks. The scenario that benefits from this strength is when relative distances between anatomical points must be accurately estimated, especially when a landmark has already been explored and exposed. For example, in head and neck tumor surgeries, surgeons often use the tragal pointer as a surgical landmark to identify the facial nerve trunk and the maxillary artery during procedures, such as parotidectomy, mandibular osteotomy, and temporomandibular joint arthroplasty [50]. In addition, in skull base surgery, surgeons often rely on stable bony landmarks, such as the occipital condyle or mastoid process, to sequentially locate cranial nerve exit points, including the jugular foramen and hypoglossal canal [51].

SI with markerless inside-out tracking and VT could be combined across different stages of the tasks. First, VT provides general spatial awareness, such as adapting to specific patient positioning, orienting with comprehensive medical imaging, or selecting approximate entry points. Once a key anatomical landmark is exposed, SI could rapidly guide surgeons to adjacent structures by using relative spatial relationships, minimizing the need for repeated attention switching [52,53]. If SI causes visual obstruction, cognitive overload, or registration instability and inaccuracy, SI can be temporarily deactivated, allowing VT to take over as a stable spatial reference. This hybrid modality enables adaptive assistance, providing surgeons with tailored support at different procedure stages based on clinical needs.

Our findings showed that user preferences were almost evenly split between SI and VT, underscoring the limitations of relying on either visualization method in isolation. Rather than competing alternatives, SI and VT could be viewed as complementary tools that respond to different scenarios. While SI enables precise overlay of subcutaneous landmarks, VT provides more reliable orientation under deformation or registration drift. These complementary features suggest that future AR systems should integrate both approaches within a single workflow.

### Limitations

This study has some limitations. First, the polystyrene foam head phantoms used in the experiment lacked realistic features, such as skin texture, natural color, and anatomical details, which are critical for accurate identification of anatomical landmarks in the real clinical scenarios. However, using these phantoms allowed for reproducible evaluation of the performance of two modalities. Second, the homogeneous and rigid phantom surface may have favored SI by registration. Unlike real surgical environments, phantoms lack deformable soft tissues, surgical draping, fluids, and light reflections, all of which can substantially increase registration and tracking errors for SI [54,55]. In contrast, VT does not require accurate overlay; thus, it was not hindered by those problems. These considerations suggest that the relative advantage of SI observed in phantom experiments may be attenuated in vivo, whereas VT could perform more robustly in real surgical settings. Third, in real clinical scenarios, the phantom’s components, such as the mandible, could not replicate the mobility of human anatomy. This mobility may pose a significant challenge to markerless inside-out registration and further accurate anatomical localization for SI. In contrast, mobile parts in VT may be a potential solution. To address these challenges, cadaver studies or studies with high-fidelity phantoms replicating the mobility of anatomical structures should be conducted to validate the clinical applicability and generalizability of the findings. Fourth, since the difference in absolute accuracy in the sample size calculation was less than 5 mm, the study may have been underpowered to detect the influence of some fixed effects. Subsequent studies should consider increasing the sample size to enhance statistical power and generalizability. Finally, current findings are constrained to the facial region, where underlying bone structures provide a stable spatial reference. It would be valuable to investigate the performance of two modalities in other regions of the body like the abdomen, where soft tissue may bring additional challenges.

### Future Directions

In addition to further validation with cadaveric studies or high-fidelity phantoms, future work should also address technical factors that directly influence localization accuracy. In particular, subcutaneous soft tissue thickness, variations in viewing perspective, and the resulting margin variability were shown to pose consistent challenges for both AR modalities. To mitigate these effects, new visualization approaches need to be developed to reduce the effects of viewing perspective and account for the effects of the overlying tissue, regardless of the visualization modality. First, the user’s viewing angle could be guided in AR. One possible strategy would be to create a virtual cylindrical tunnel of 2 circles between the target structure and the skin surface, orienting the user to view in a planned direction. Second, the AR visualization should establish a clear connection between the overlying tissue and the target structures, for example, for nerve exit points, a line connecting the points and their planned skin projection, clearly identifying the planned margin and mitigating the inaccuracy introduced by the overlying tissue.

While these approaches address specific visualization challenges, the next step lies in advancing toward a hybrid, context-aware AR system. With advances in registration accuracy, hardware performance, and integration of AI technologies, such a system could autonomously detect procedural phases, surgical context, and anatomical exposure. Based on this contextual understanding, it could dynamically switch between VT and SI modes, providing global spatial orientation and reference by VT and precise overlays for local structure localization by SI. This intelligent modality would reduce cognitive load and enable phase-specific surgical guidance.

### Conclusion

This study systematically compared SI with markerless inside-out tracking and VT for surgical localization tasks in the head and neck region. SI demonstrated superior localization accuracy in 0D structures, whereas VT revealed robust spatial orientation, comparable accuracy in 2D, and superior accuracy in 3D structures. These complementary strengths suggested that VT represents a viable alternative for macro localization, while SI may be preferable for fine-grained, sequential landmark tasks. Rather than assuming SI to be universally applicable across all surgical contexts, our findings emphasize the need for context-adaptive AR strategies that can dynamically leverage the strengths of both modalities.

## Supplementary material

10.2196/75962 Checklist 1 CONSORT checklist.

## Abbreviations

AR: augmented reality
ASD: average surface distance
CONSORT: Consolidated Standards of Reporting Trials
CT: computed tomography
HD: Hausdorff distance
HL2: HoloLens 2
LMM: linear mixed-effects model
NASA-TLX: National Aeronautics and Space Administration Task Load Index
RWTH: Rheinisch-Westfälische Technische Hochschule
SNS: surgical navigation systems
VI-SLAM: visual inertial-simultaneous localization and mapping
VT: virtual twin
